# Identification and denitrification characteristics of a salt-tolerant denitrifying bacterium *Pannonibacter phragmitetus* F1

**DOI:** 10.1186/s13568-019-0918-y

**Published:** 2019-12-03

**Authors:** Xinyi Wang, Hui Zhu, Brian Shutes, Baorong Fu, Baixing Yan, Xiangfei Yu, Huiyang Wen, Xin Chen

**Affiliations:** 1 Key Laboratory of Wetland Ecology and Environment, Northeast Institute of Geography and Agroecology, Chinese Academy of Sciences, Changchun, 130102 People’s Republic of China; 2 Jilin Provincial Engineering Center of CWs Design in Cold Region & Beautiful Country Construction, Changchun, 130102 People’s Republic of China; 3 School of Environment, Liaoning University, Shenyang, 110036 People’s Republic of China; 4 Urban Pollution Research Centre, Middlesex University, Hendon, London, NW4 4BT UK

**Keywords:** Denitrifying bacteria, Salt tolerance, Denitrification, *Pannonibacter phragmitetus*

## Abstract

A salt-tolerant denitrifying bacterium F1 was isolated in this study, which has high nitrite (NO_2_^−^–N) and nitrate (NO_3_^−^–N) removal abilities. The salt tolerance capacity of strain F1 was further verified and the effects of initial pH, initial NaNO_2_ concentration and inoculation size on the denitrification capacity of strain F1 under saline conditions were evaluated. Strain F1 was identified as *Pannonibacter phragmitetus* and named *Pannonibacter phragmitetus* F1. This strain can tolerate NaCl concentrations up to 70 g/L, and its most efficient denitrification capacity was observed at NaCl concentrations of 0–10 g/L. Under non-saline condition, the removal percentages of NO_2_^−^–N and NO_3_^−^–N by strain *Pannonibacter phragmitetus* F1 at pH of 10 and inoculation size of 5% were 100% and 83%, respectively, after cultivation for 5 days. Gas generation was observed during the cultivation, indicating that an efficient denitrification performance was achieved. When pH was 10 and the inoculation size was 5%, both the highest removal percentages of NO_2_^−^–N (99%) and NO_3_^−^–N (95%) by strain *Pannonibacter phragmitetus* F1 were observed at NaCl concentration of 10 g/L. When the NaCl concentration was 10 g/L, strain *Pannonibacter phragmitetus* F1 can adapt to a wide range of neutral and alkaline environments (pH of 7–10) and is highly tolerant of NaNO_2_ concentration (0.4–1.6 g/L). In conclusion, strain *Pannonibacter phragmitetus* F1 has a great potential to be applied in the treatment of saline wastewater containing high nitrogen concentrations, e.g. coastal aquaculture wastewater.

## Introduction

Due to the advantages of a short production cycle, high yield and convenient management, coastal aquaculture has rapidly developed as a pillar industry in the coastal region of China (Liang et al. 2018). For semi-intensive and/or intensive mariculture, the feed utilization rates were only about 4.0–27.4% (Chen et al. 2016). Owing to lack of indispensable production management and pollution control, more than 60% of the nitrogen in feed for coastal aquaculture was discharged into the adjacent waters without sufficient treatment, resulting in severe eutrophication and deterioration of ecological quality in coastal watersheds (Chen et al. 2016; Kroupova et al. 2005). A high concentration of nitrate can lead to water eutrophication, and even impose a serious threat to human health (e.g., malformation, cancerization, and mutation, etc.) when it is transformed into nitrosamines (Bhatnagar and Sillanpaa 2011; Zhao et al. 2011). High concentrations of nitrite can cause the suffocation of aquatic animals (e.g., fish, shrimp, etc.) by oxidizing the Fe(II) in hemoglobin and generating methemoglobin which would restrain the combination and release of hemoglobin towards oxygen (Zhang et al. 2018). Therefore, the development of an applicable technology for in situ removal of nitrate nitrogen (NO_3_^−^–N) and nitrite nitrogen (NO_2_^−^–N) in wastewater originating from coastal aquaculture is of urgent needed.

Microbial denitrification has many advantages, e.g., high efficiency, low economical investment, easy operation, and no secondary pollution, etc., and is consequently widely used to treat nitrogen contaminated water. Nitrogen removal by microorganisms mainly uses bacteria (e.g. nitrifying bacteria, denitrifying bacteria, etc.) with nitration and denitrification capacities to achieve morphological transformation of various nitrogenous compounds in wastewater (Lv et al. 2017). Most of NO_2_^−^–N in wastewater is removed by the nitrification of nitrifying bacteria. However, due to the slow growth of nitrifying bacteria, and they must grow in aerobic conditions, a long time is needed for their cultivation (Spieck and Lipski 2011; Vekeman et al. 2013). Therefore, it is difficult for nitrifying bacteria to become dominant bacteria and play their role in practical applications. Additionally, although the nitrification of nitrifying bacteria can effectively remove NO_2_^−^–N from water, it cannot thoroughly solve the nitrogen pollution problem, as nitrate is the final product of microbial nitrification and the denitrification of NO_3_^−^–N is still needed. Denitrifying bacteria, however, can make up for the limitation of nitrifying bacteria. In anoxic and anaerobic conditions, the NO_2_^−^–N and NO_3_^−^–N in wastewater can be transformed by denitrifying bacteria into nitrogen (N_2_) and nitrous oxide (N_2_O), which are eventually released into the atmosphere, and greatly reduce the nitrogen concentration in water (Coban et al. 2015). Coastal aquaculture wastewater contains not only high concentrations of nitrogen, but also a large amount of soluble salts. In a high saline environment, the growth and metabolism of most bacteria are likely to be inhibited, and even lead to death. This is mainly due to the increase of osmotic pressure caused by high salinity, the destruction of bacterial cell membranes, the excessive loss of water in bacterial cells and the separation of protoplasm (Liu et al. 2009). High salinity leads to the limitation of denitrification capacity for general denitrifying bacteria (Tang et al. 2014). Therefore, to identify bacteria with both denitrification capacity and salt tolerant characteristic is of great practical significance.

Many studies have been conducted to remove nitrogen from wastewater by denitrifying bacteria. For example, a heterotrophic nitrification bacterium *Klebsiella* sp. TN-10 was isolated from tannery wastewater, and it exhibited excellent characteristics to remove both NO_3_^−^–N and NO_2_^−^–N, with the removal percentages of 95% and 100%, respectively (Li et al. 2019). A novel heterotrophic bacterium *Acinetobacter* sp. T1 was isolated from activated sludge of a pig farm wastewater treatment plant and exhibited efficient heterotrophic nitrification and aerobic denitrification capability to utilize ammonium (NH_4_^+^–N), NO_3_^−^–N or NO_2_^−^–N as the sole nitrogen source, and the removal rates of 12.08, 5.53 and 1.69 mg/L/h, were obtained for NH_4_^+^–N, NO_3_^−^–N, and NO_2_^−^–N, respectively (Chen et al. 2019). Although extensive research has been carried out for removing nitrogen by using denitrifying bacteria, there are, however, only a few studies adequately cover the research and/or application of salt-tolerant denitrifying bacteria with salt-tolerance characteristics and efficient denitrification performance. A novel halophilic bacterium *Bacillus litoralis* N31 was isolated from mariculture water, and its nitrification rate increased with increasing initial NH_4_^+^–N concentration (from 10 to 250 mg/L) at 30–40 g/L NaCl (Huang et al. 2017). However, the removal ability of the strain *Bacillus litoralis* N31 to NO_2_^−^–N and NO_3_^−^–N was not verified. In summary, salt-tolerant nitrite-type denitrifying bacteria are necessary to be isolated and screened to treat coastal aquaculture wastewater with high nitrite concentration.

Therefore, in order to fill this technology gap, this study aims to: (1) identify the strain F1 that was obtained from seawall muddy water; (2) evaluate the denitrification capacity and salt tolerance of strain F1; and (3) quantify the effects of initial pH, initial NaNO_2_ concentration and inoculation size on the denitrification capacity of strain F1 under saline conditions. This study can provide an alternative and effective microbial resource for nitrogen removal in saline wastewater. Furthermore, this study may also promote the development of bioremediation technology for water quality control in coastal aquaculture systems.

## Materials and methods

### Bacterial strain and media

Strain F1 was isolated and screened from the seawall muddy water samples in Dalian City, Liaoning Province (39° 38′ 31′′ N, 122° 58′ 19′′ E), and stored in the Key Laboratory of Wetland Ecology and Environment, Chinese Academy of Sciences, China.

The culture media was described as follows: (1) ingredients for the denitrification medium (pH = 10) were: CH_3_COONa 5 g, K_2_HPO_4_ 1 g, NaNO_2_ 0.8 g (The initial nitrogen concentration was approximate 160 mg/L, including 115.9 ± 4.63 mg/L of NO_2_^−^–N and 42.47 ± 1.19 mg/L of NO_3_^−^–N, respectively. The NO_3_^−^–N was generated by oxidation of NO_2_^−^–N during medium preparation), CaCl_2_ 0.03 g, NaCO_3_ 1 g, FeSO_4_·7H_2_O 0.06 g, MgSO_4_·7H_2_O 0.2 g, deionized water 1000 mL. It is worth noting that FeSO_4_·7H_2_O was added after the addition of deionized water to avoid oxidation of divalent iron (Fe^2+^) to ferric iron (Fe^3+^) (Wu et al. 2019); (2) ingredients for the screen solid plate medium (pH = 10) were the same as the denitrification medium but with 2% agar (m/v) added. All media were autoclaved at 121 °C for 30 min before being used.

### Identification of strain F1

#### Colony morphology and physiological and biochemical experiments

According to *Bergey’s manual of systematic bacteriology* (Goodfellow et al. 2012), the colony morphology and the physiological and biochemical characteristics of strain F1 were studied.

#### 16S rDNA sequencing

The total DNA of strain F1 genome was extracted by a conventional method using an Ezup column bacterial genomic DNA extraction kit (Sangon, China). The polymerase chain reaction (PCR) amplification of strain F1 genomic DNA was performed using total DNA as a template. The PCR reaction primer was a 16S rDNA amplification universal primer, in which the forward primer was 27F 5′-AGAGTTTGATCCTGGCTCAG-3′ and the reverse primer was 1492R 5′-GGTTACCTTGTTACGACTT-3′. The PCR reaction process was carried out in a 25 μL system. The composition of the reaction system was described as follows: DNA template 0.5 μL, dNTP (mix) 1 μL, 10× Buffer (with Mg^2+^) 2.5 μL, Taq enzyme 0.2 μL, primer F (10 μM) 0.5 μL, primer R (10 μM) 0.5 μL, and double distilled water to 25 μL. PCR amplification was completed under the following conditions: initial denaturation at 94 °C for 4 min; 30 cycles of 94 °C for 45 s, 55 °C for 45 s, and 72 °C for 60 s, and a final extension at 72 °C for 10 min, and termination at 4 °C. A total of 5 μL of the PCR amplification product was taken and detected by 1% agarose gel electrophoresis. After verification, the strip was cut and the PCR product was purified using a SanPrep column DNA Jgel recovery kit (Sangon, China). The recovered PCR products were sent to Sangon Biotech (Shanghai) company limited in China for 16S rDNA sequencing.

#### Phylogenetic analysis

The partial sequences obtained by 16S rDNA sequencing were searched against GenBank using the Advanced BLAST similarity search option accessible from the homepage at the National Center for Biotechnology Information (NCBI) (http://www.ncbi.nlm.nih.gov/). The phylogenetic analysis was performed by using Mega 4.0 version (Arizona State University, 2007) software to search for related sequences with high homology. Multiple sequence alignment analysis was performed with program Clustal X. The evolutionary status of the phylogenetic tree of the bacteria in similar strains was analyzed by Neighbor-Joining (N-J) algorithm. The stability of the phylogenetic tree was analyzed by Boot-strap and evaluated based on 1000 replicates.

### Experiments on salt-tolerance and nitrogen removal characteristics of strain F1

#### Denitrification capacity test

The culture solution of strain F1 with 5% inoculation size (v/v) was placed in 250 mL conical flask that contained 200 mL of sterilized denitrification medium to create an anoxic environment, and was cultivated in a constant temperature incubator at 30 °C for 5 days. The concentrations of NO_2_^−^–N and NO_3_^−^–N in the denitrification medium were determined daily and the denitrification performance of strain F1 was evaluated. Three independent parallel experiments were designed for each treatment.

#### Salt tolerance test

The growth of strain F1 was observed by changing the salinity levels of the screen solid plate medium. The salinity levels were set at 0, 30, 50, 70, and 100 g/L NaCl concentrations, respectively. Strain F1 was streaked into the medium with each respective salinity treatment, and cultivated in a constant temperature incubator at 30 °C for 5 days. The bacterial colony growth was regarded as the basis for determining whether strain F1 tolerate the corresponding salinity levels. Three independent parallel experiments were designed for each treatment.

### Effect of salinity levels on denitrification capacity of strain F1

The salinity levels of denitrification medium were set at 0, 10, 30, 50, 70, and 100 g/L NaCl concentrations, respectively. Strain F1 was transferred to each respective sterilized saline denitrification medium at a 5% inoculation size (v/v), and the mouth of the conical flask was sealed and statically cultured at 30 °C for 5 days. The concentrations of NO_2_^−^–N and NO_3_^−^–N in the culture medium were determined before and after the 5 days cultivation and the denitrification performance of strain F1 was evaluated. Three independent parallel experiments were designed for each treatment.

### Effect of initial pH value, initial NaNO_2_ concentration, and inoculation size on denitrification capacity of strain F1 in saline condition

A 200 mL sterilized denitrification medium containing 10 g/L NaCl concentration was packed in a 250 mL conical flask and strain F1 was cultivated in a sealed static chamber at 30 °C for 5 days. The NO_2_^−^–N and NO_3_^−^–N removal abilities of strain F1 were tested as affected by initial pH value, initial NaNO_2_ concentration, and inoculation size, respectively. (1) The initial pH values were set at 3, 5, 7, 9, 10, and 11, respectively; (2) the initial NaNO_2_ concentrations were set at 0.4, 0.8, 1.6, 2.4, and 3.2 g/L (NaNO_2_ was added into the denitrifying medium without any initial NaNO_2_), respectively; and (3) the inoculation sizes (v/v) were set at 1, 3, 5, 7, and 10%, respectively. After a 5 days static cultivation, the concentrations of NO_2_^−^–N and NO_3_^−^–N in the medium were determined. In this experiment, only each respective factor tested was changed while the other conditions remained constant. Three independent parallel experiments were designed for each treatment.

### Sample analysis

The culture solution was centrifuged at 5000 r/min for 10 min, and the bacteria were separated. The supernatant was then diluted for measurement. The concentrations of NO_2_^−^–N and NO_3_^−^–N in water samples were determined by N-(1-naphthyl)-ethylenediamine spectrophotometry and naphthylethylenediamine hydrochloride spectrophotometry, respectively.

### Statistical analysis

Statistical analysis of all the data was performed by using Microsoft Office Excel 2007 software and SPSS Statistics 22.0. All graphs were prepared by using Origin 9.1 (USA) software. The data in all figures was expressed as mean ± standard deviation. The means between different treatments were compared by one-way ANOVA with Tukey’s test at a significance level of 0.05.

The Genbank registration number of strain F1 was MN396535.

## Results

### Identification of strain F1

#### Microbial morphology and the physiological and biochemical characteristics of strain F1

Strain F1 was cultivated in the screen solid plate medium, and the colonies grown were round, small, neat edges, smooth surface and luster. Under the optical microscope, the bacteria morphology of strain F1 was observed to be rod-shaped and confirmed to belong to gram-negative bacteria by gram staining. The physiological and biochemical characteristics of strain F1 are shown in Table 1.

**Table 1:** Physiological and biochemical characteristics of strain F1

Characteristic	Result	Characteristic	Result
Metabolism	Facultative anaerobic	Glucose oxidative fermentation	Fermentation
Gram’s stain	–	Catalase	+
1% NaCl	+	Oxidase	+
3% NaCl	+	Gelatin hydrolysis	–
5% NaCl	+	H_2_S test	+
Nitrate reduction	+	V-P test	+
Denitrification	+	M.R test	+

“+” means positive and growth, “−” means negative and no growth

#### 16S rDNA gene sequence and phylogenetic analysis

**Fig. 1:**
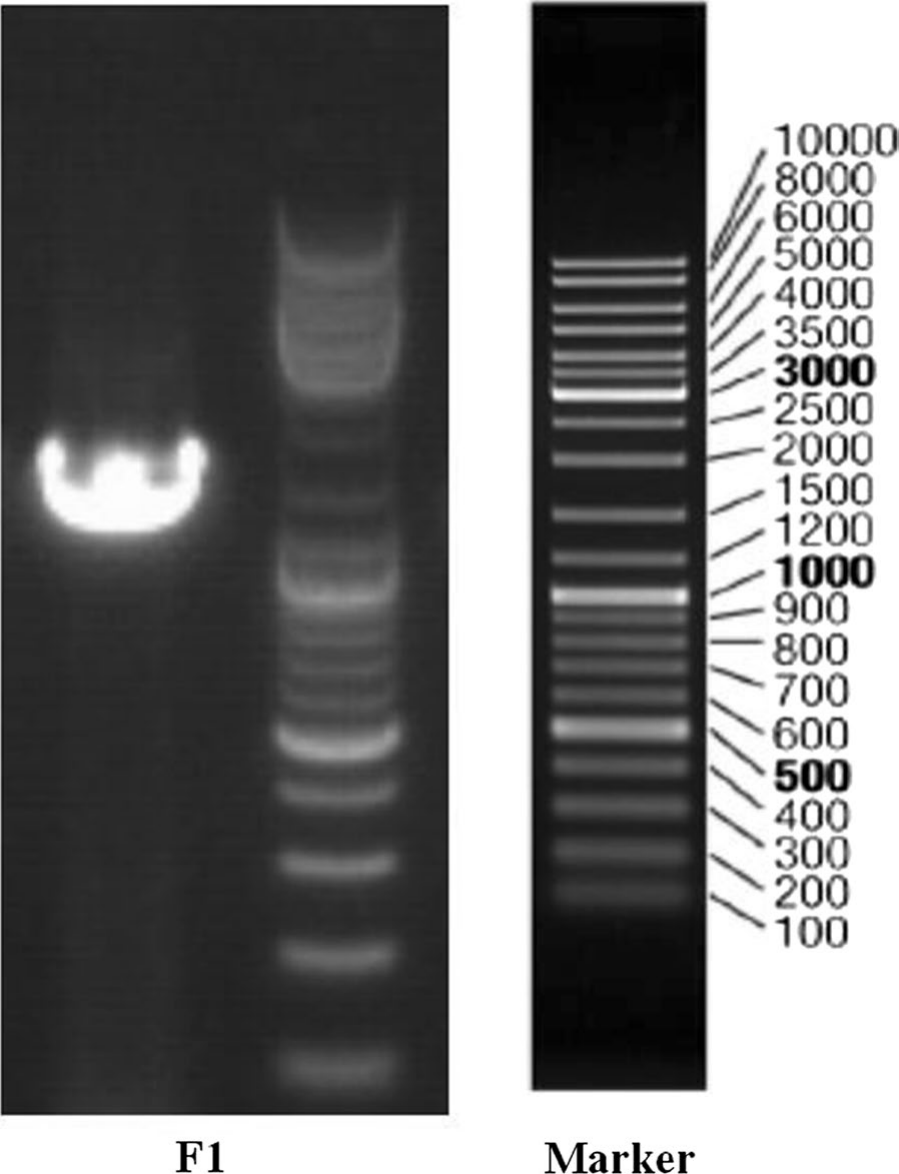
Amplification of 16S rDNA gene of strain F1 by PCR

**Fig. 2:**
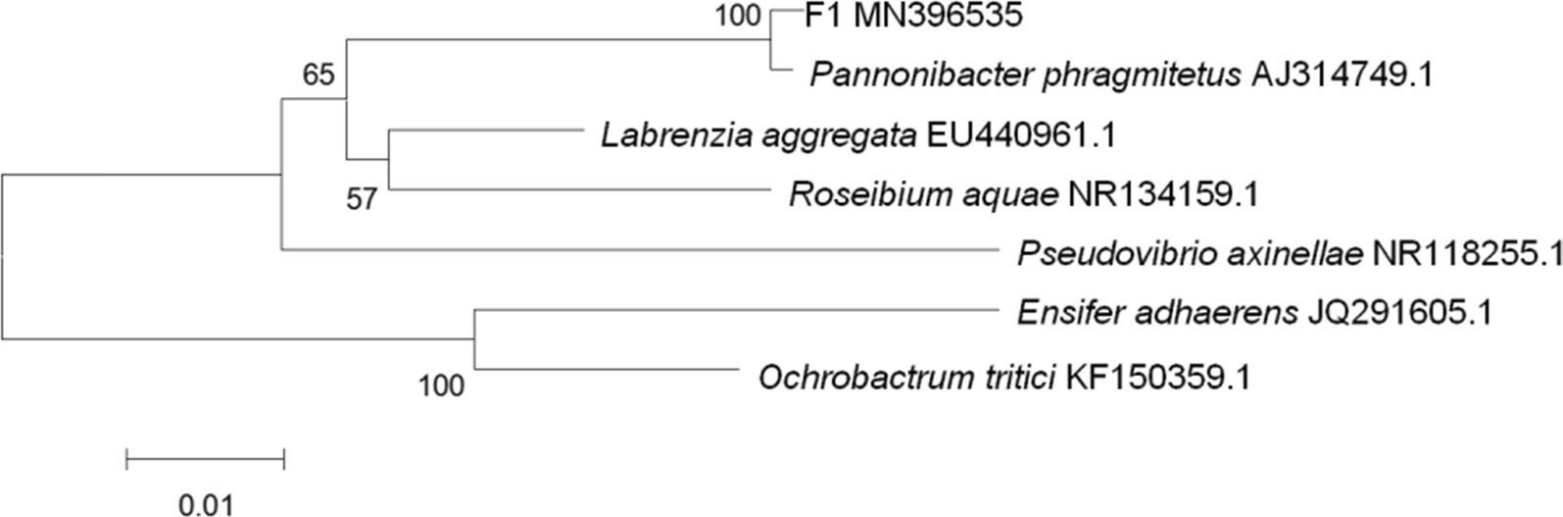
Phylogenestraintic tree map of 16S rDNA gene sequence constructed by strain F1 and similar strains

Strain F1 was identified by a series of DNA extraction, PCR amplification and agarose gel electrophoresis. The full length of the16S rDNA gene of strain F1 was 1440 bp (Fig. 1). A phylogenetic tree constructed by MEGA 4.0 showed that strain F1 was closest to the phylogenetic status of *Pannonibacter phragmitetus* (Fig. 2). The strain F1 was closest to the phylogenetic status of *Pannonibacter phragmitetus*. The 16S rDNA identification reveals that strain F1 is 99.72% homology genetic relationship with *Pannonibacter phragmitetus* (Genbank registration number: AJ314749.1). Therefore, according to morphological observation, the physiological and biochemical characteristics of strains, and 16S rDNA gene analysis, strain F1 was identified as *Pannonibacter phragmitetus* and named *Pannonibacter phragmitetus* F1. It was preserved at CGMCC on March 25, 2019, numbered CGMCC No: 17432.

### Denitrification capacity of strain F1

**Fig. 3:**
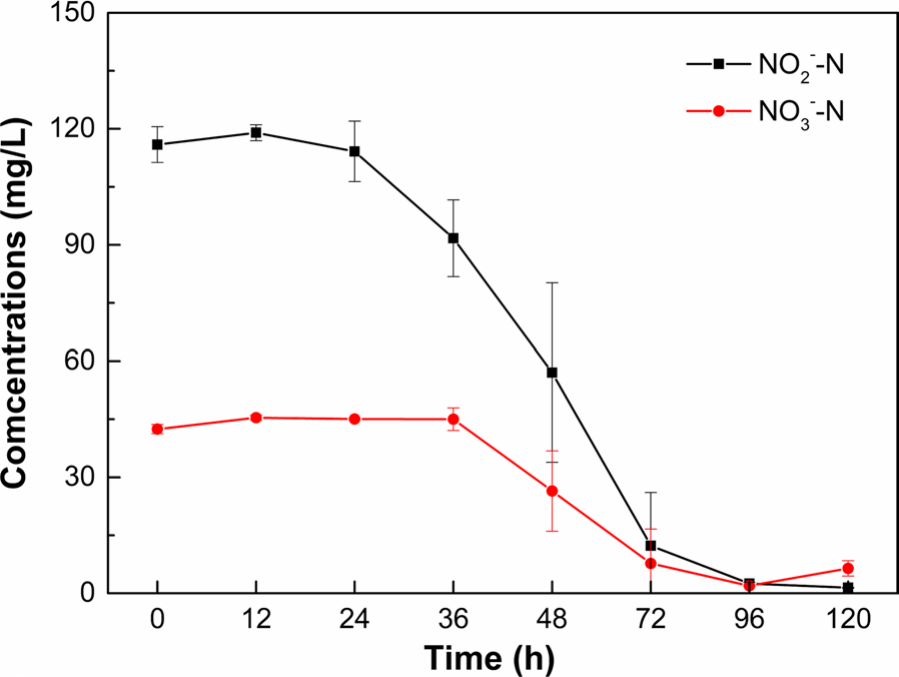
Removal of NO_2_^−^–N and NO_3_^−^–N by strain F1 in denitrification medium with different NaCl concentrations. Values represent the mean of three replicates and error bars represent standard deviations. Columns containing different letters indicate significant differences among treatments at *p *< 0.05 by Tukey’s test

NaNO_2_ was used as the sole source of nitrogen in this experiment. A small amount of NaNO_2_ however was still oxidized and rapidly converted into NaNO_3_, as reported by (Philips et al. 2002). Therefore, the initial nitrogen source of the denitrification medium included 115.9 ± 4.63 mg/L of NO_2_^−^–N and 42.47 ± 1.19 mg/L of NO_3_^−^–N, respectively. As shown in Fig. 3, the concentration of NO_2_^−^–N in the denitrification medium maintained stable in the prime 24 h statically cultivation, thereafter, the concentration of NO_2_^−^–N decreased with increasing cultivation time. For NO_3_^−^–N, however, the concentration remained stable until 36 h after cultivation, and the concentration of NO_3_^−^–N then decreased with increasing cultivation time. In general, the decrease of NO_2_^−^–N concentration was faster than NO_3_^−^–N, as a bigger slope was observed for NO_2_^−^–N than NO_3_^−^–N. After cultivation of 96 h, both removal percentages of NO_2_^−^–N and NO_3_^−^–N by strain F1 were greater than 95%, i.e., the concentrations of NO_2_^−^–N and NO_3_^−^–N decreased from 115.90 ± 4.63 mg/L to 2.50 ± 0.33 mg/L, and from 42.47 ± 1.19 mg/L to 1.93 ± 0.21 mg/L, with the corresponding removal percentages of 98% and 95%, respectively. While, with the further increasing of cultivation time (i.e., 120 h), both concentrations of NO_2_^−^–N and NO_3_^−^–N remained relatively stable. It is noteworthy that during the course of cultivation, the decrease of NO_2_^−^–N and NO_3_^−^–N concentrations was accompanied by gas formation, indicating that NO_2_^−^–N and NO_3_^−^–N in the denitrification medium were transformed into gases by strain F1. For the control treatment without inoculating strain F1, the concentrations of NO_2_^−^–N and NO_3_^−^–N did not evidently change during the cultivation process (only 4% nitrite was transformed to nitrate).

### Salt tolerance of strain F1

Strain F1 was screened from seawall muddy water, which was considered a saline environment. Therefore, we speculate that strain F1 is salt tolerant, to some degree. To verify this speculation, strain F1 was cultivated in screen solid plate medium with different salinity treatments for 5 days. The growth of strain F1 under different salinity treatments with increasing cultivation time is shown in Table 2. There were no colonies observed in medium with any salinity treatments after cultivation for 1 day. After cultivation for 2 days, the colonies started to grow obviously in medium with 0 and 30 g/L NaCl concentrations, and trace colonies was observed to grow in 50 g/L NaCl concentrations as well, but there were no colonies grew in medium with 70 and 100 g/L NaCl concentrations. After cultivation for 3 days, obvious colonies appeared in the medium with 0, 30, and 50 g/L NaCl concentrations, and trace colonies grew in the medium with 70 g/L NaCl concentration. After cultivation for 5 days, colony growth was observed in the medium with 0, 30, 50, and 70 g/L NaCl concentrations. There was no colony growth observed in the medium with 100 g/L NaCl concentration throughout the experiment. The above observation indicates that strain F1 can tolerate a wide range of salinity levels with up to 70 g/L NaCl concentration, although 70 g/L NaCl treatment lead to a slower growth rate of strain F1 compared to lower salinity treatments. Extremely high salinity treatment, i.e., 100 g/L NaCl concentration in this study, however, stopped the growth of strain F1.

**Table 2:** Growth of strain F1 in screen solid plate medium with different salinity treatments

Cultivate time (days)	NaCl concentration in screen solid plate medium (g/L)				
	0	30	50	70	100
1	–	–	–	–	–
2	++	++	+	–	–
3	++	++	++	+	–
4	++	++	++	+	–
5	++	++	++	++	–

++: Obvious colonies appeared on the surface of the medium, indicating that strain F1 grew normally

+: A small number of colonies appeared on the surface of the medium, indicating that strain F1 grew but with low growth rate

−: No colonies appeared on the surface of the medium, indicating that strain F1 did not grow

### Effect of salinity on denitrification capacity of strain F1

**Fig. 4:**
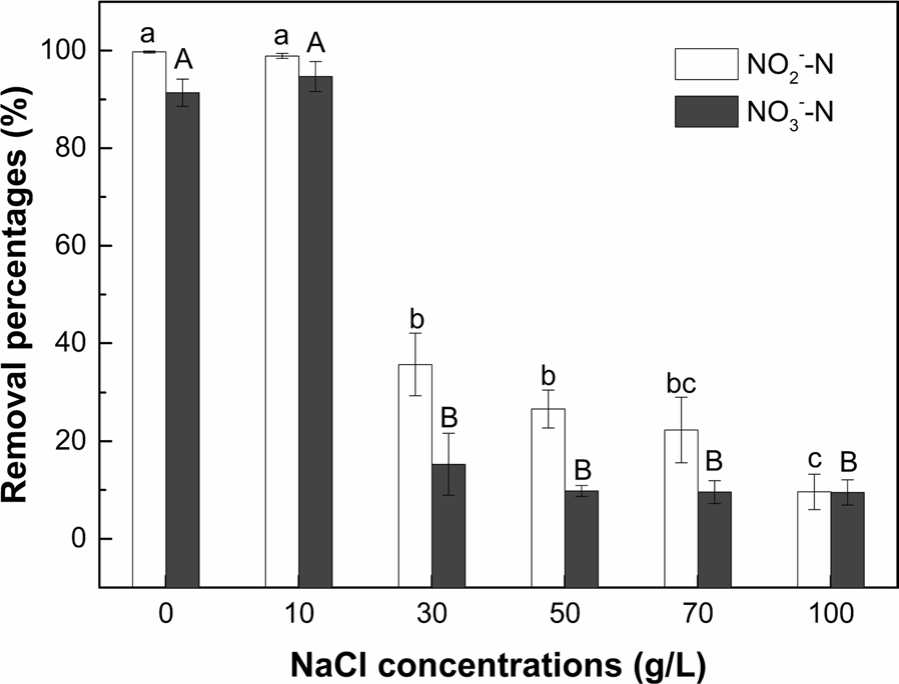
Removal of NO_2_^−^–N and NO_3_^−^–N by strain F1 in denitrification medium with different NaCl concentrations. Values represent the mean of three replicates and error bars represent standard deviations. Columns containing different letters indicate significant differences among treatments at *p *< 0.05 by Tukey’s test

The removal percentages of NO_2_^−^–N and NO_3_^−^–N of strain F1 under different salinity levels after a 5 days cultivation are presented in Fig. 4. Strain F1 maintained high removal percentages for NO_2_^−^–N (99–100%) and NO_3_^−^–N (91–95%) when the NaCl concentration was below 10 g/L. However, the removal ability of strain F1 to NO_2_^−^–N and NO_3_^−^–N was significantly (*p* < 0.05) inhibited when the NaCl concentration was increased to 30 g/L and above. In the medium with 30, 50, 70 and 100 g/L NaCl concentrations, strain F1 exhibited low removal percentages of NO_2_^−^–N and NO_3_^−^–N, with the NO_2_^−^–N and NO_3_^−^–N removal percentages of 10–36% and 10–15%, respectively. This observation indicates that the NaCl concentration of 30 g/L and above had a significant (*p* < 0.05) and negative impact on the denitrification capacity of strain F1 compared to 0 and 10 g/L NaCl treatments. There was no significant difference in the NO_3_^−^–N removal percentage among treatments with 30, 50, 70 and 100 g/L NaCl concentrations. However, the NO_2_^−^–N removal percentage by strain F1 in treatment of 100 g/L NaCl concentrations was significantly (*p* < 0.05) lower than in 30 and 50 g/L NaCl concentrations.

### Effects of various factors on the denitrification capacity of strain F1 in saline conditions

#### Initial pH

**Fig. 5:**
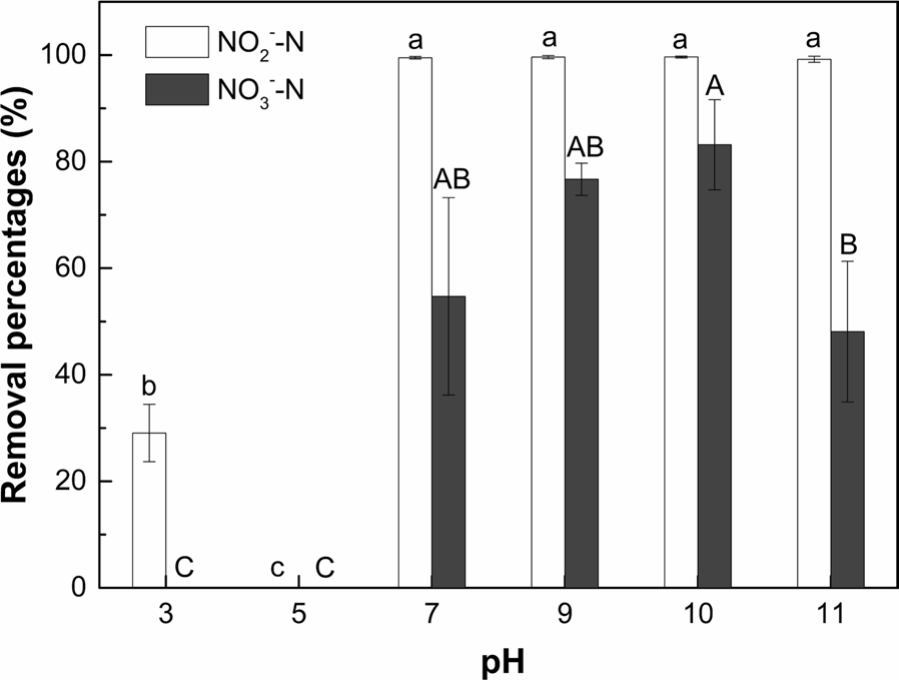
Effect of initial pH on denitrification capacity of strain F1 in saline condition (10 g/L NaCl). Values represent the mean of three replicates and error bars represent standard deviations. Columns containing different letters indicate significant differences among treatments at *p *< 0.05 by Tukey’s test

Figure 5 illustrates the removal percentages of NO_2_^−^–N and NO_3_^−^–N by strain F1 in saline conditions (10 g/L NaCl) under different initial pH after cultivation for 5 days. The pH is one of the main external factors that can both directly and indirectly affect the growth and metabolism of microorganisms. Therefore, it is particularly important to evaluate the effect of initial pH on the denitrification capacity of denitrifying bacteria, i.e., strain F1 in this study. As shown in Fig. 5, under acidic condition (i.e., pH of 3 and 5), NO_3_^−^–N was not removed by strain F1. While, strain F1 exhibited a low NO_2_^−^–N removal ability (removal percentage of 29%) at the pH = 3. When the pH was increased to 7–11, the mean removal percentages of NO_2_^−^–N and NO_3_^−^–N by strain F1 were above 99% and above 48%, respectively. There was no significant difference in NO_2_^−^–N removal percentages among treatments with pH of 7, 9, 10, and 11. For NO_3_^−^–N, the removal percentage of NO_3_^−^–N reached a maximum value (83%) when the pH was 10, and the removal percentage was significantly (*p* < 0.05) higher than pH of 11. The above observation demonstrates that strain F1 could adapt to a wide range of neutral and alkaline environments (i.e., pH of 7–11) under saline conditions (i.e., 10 g/L NaCl).

#### Initial NaNO_2_ concentration

**Fig. 6:**
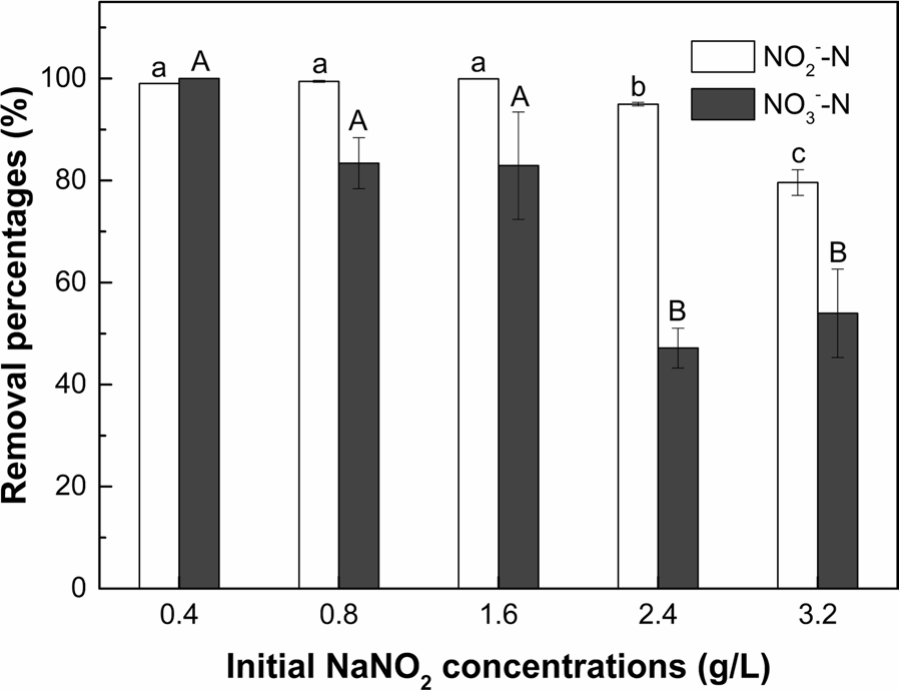
Effect of initial NaNO_2_ concentration on denitrification capacity of strain F1 in saline condition (10 g/L NaCl). Values represent the mean of three replicates and error bars represent standard deviations. Columns containing different letters indicate significant differences among treatments at *p *< 0.05 by Tukey’s test

Figure 6 shows the removal percentages of NO_2_^−^–N and NO_3_^−^–N by strain F1 in saline condition (10 g/L NaCl) under different initial NaNO_2_ concentrations after cultivation for 5 days. The greatest removal percentages of NO_2_^−^–N (99%) and NO_3_^−^–N (100%) by strain F1 were observed when the initial NaNO_2_ concentration was 0.4 g/L. When the initial NaNO_2_ concentrations were 0.8–1.6 g/L, the removal percentages of NO_2_^−^–N and NO_3_^−^–N by strain F1 were maintained at approximately 99% and 83%, respectively. With the increasing initial NaNO_2_ concentration (i.e., 2.4 and 3.2 g/L), the removal percentages of both NO_2_^−^–N and NO_3_^−^–N by strain F1 significantly declined (*p* < 0.05). However, strain F1 still exhibited a promising NO_2_^−^–N removal capacity (removal percentage of 80%) when the NaNO_2_ concentration was 3.2 g/L. The removal percentages of NO_3_^−^–N by strain F1 in treatments of 2.4 and 3.2 g/L initial NaNO_2_ concentration (47% and 54%, respectively) were significantly (*p* < 0.05) lower than 0.4, 0.8, and 1.6 g/L NaNO_2_ treatments (100%, 83% and 83%, respectively). Although the removal percentages of NO_2_^−^–N and NO_3_^−^–N by strain F1 decreased with the increasing initial NaNO_2_ concentration, strain F1 still exhibited some capacity for NO_2_^−^–N and NO_3_^−^–N removal in high initial NaNO_2_ concentration treatments. In general, strain F1 can tolerate a high concentration of NaNO_2_ up to 3.2 g/L.

#### Inoculation size

**Fig. 7:**
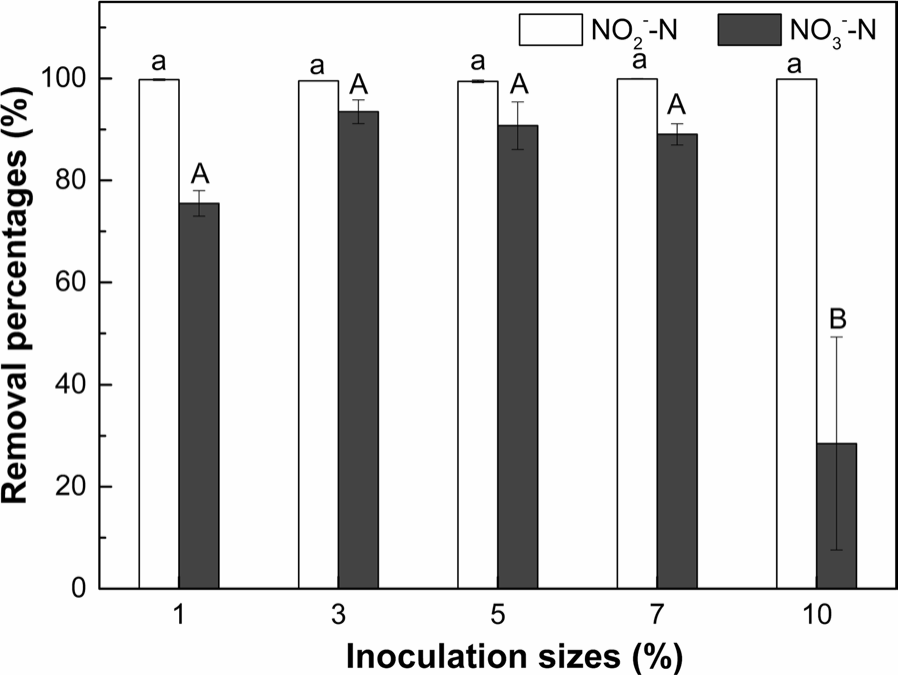
Effect of inoculation size on denitrification capacity of strain F1 in saline condition (10 g/L NaCl). Values represent the mean of three replicates and error bars represent standard deviations. Columns containing different letters indicate significant differences among treatments at *p *< 0.05 by Tukey’s test

The removal percentages of NO_2_^−^–N and NO_3_^−^–N by strain F1 in saline conditions (10 g/L NaCl) under different inoculation sizes after cultivation for 5 days are presented in Fig. 7. The NO_2_^−^–N removal percentage by strain F1 in denitrification medium was higher than 99% for all the tested inoculation sizes. However, the removal percentage of NO_3_^−^–N was significantly (*p* < 0.05) reduced by the inoculation size of 10% compared to other treatments. When the inoculation size was 1–7%, the removal percentages of NO_3_^−^–N by strain F1 in the denitrification medium were between 75 and 93%. However, the removal percentage of NO_3_^−^–N was only 28% when the inoculation size was increased to 10%. This observation indicates that extremely high inoculation size (i.e., 10% in this study) can reduce the denitrification capacity of strain F1.

## Discussion

### Salt-tolerance and denitrification capacity of the strain *Pannonibacter phragmitetus* F1 as compared to similar strains of *Pannonibacter phragmitetus*

A few similar strains of *Pannonibacter phragmitetus* were isolated and most of the related studies were conducted regarding their ability to reduce Cr(VI) in soil with high Cr(VI) concentrations, to detoxify polycyclic aromatic hydrocarbons under extreme conditions and their drug resistance to elucidate antibiotic resistance and infectivity mechanisms etc. (Zhou et al. 2017; Chai et al. 2019). To the best of our knowledge, only one study was conducted to evaluate the denitrification capacity of similar strains of *Pannonibacter phragmitetus*. A heterotrophic denitrifying bacterium *Pannonibacter phragmitetus* B1 was isolated from aquaculture water, which had denitrifying genes *nirK*, *norB* and *narG* (Bai et al. 2019). Strain *Pannonibacter phragmitetus* B1 was proved to have a good denitrification capacity, with the denitrification percentages of 99%, 100% and 99%, for NH_4_Cl, NaNO_2_ and NaNO_3_, respectively, when using NH_4_Cl, NaNO_2_, and NaNO_3_ as sole nitrogen source and the initial concentration of each respective nitrogen species was 14 mg/L (Bai et al. 2019). The salt-tolerance of strain *Pannonibacter phragmitetus* B1, however, was not investigated, which hindered its application in saline conditions.

In this study, the salt tolerance of strain *Pannonibacter phragmitetus* F1 was verified after confirming its denitrification capacity, which fills both the knowledge and technical gaps of application in saline conditions. Strain *Pannonibacter phragmitetus* F1 was proved to be an efficient salt-tolerant strain, which can tolerate NaCl concentrations of up to 70 g/L. When the NaCl concentration was below 10 g/L, strain *Pannonibacter phragmitetus* F1 exhibited promising NO_2_^−^–N and NO_3_^−^–N removal efficiencies, with the removal percentages as high as that in non-saline conditions. Additionally, although strain *Pannonibacter phragmitetus* F1 showed a slower growth rate in saline conditions of 30–70 g/L NaCl concentrations, it still exhibited denitrification ability (i.e., the NO_2_^−^–N removal percentages of 23–36%). Salinity levels of coastal aquaculture wastewater vary with the location of aquaculture ponds (e.g., intertidal zone and supratidal zone, etc.) and aquaculture species (e.g., brackish water, brackish water and broad-salt species, etc.) (Primavera 2006). The salinity levels of coastal aquaculture wastewater reportedly ranged from 2 to 35 g/L NaCl concentrations (McIntosh and Fitzsimmons 2003; Tho et al. 2014; Li et al. 2018). The salt-tolerance of strain *Pannonibacter phragmitetus* F1 in this study was within the range of coastal aquaculture water. The high salt-tolerance and promising denitrification capacity of strain *Pannonibacter phragmitetus* F1 observed in this study provide a great potential for practical application in coastal aquaculture wastewater treatment. It is noteworthy that the salt tolerance of strain *Pannonibacter phragmitetus* F1 is currently domesticated in our laboratory for further improving its denitrification performance in higher salinity conditions, thereby extending its range of application.

### Tolerance of strain *Pannonibacter phragmitetus* F1 to nitrite

As far as we know, most denitrifying bacteria were screened using nitrate as sole nitrogen source, e.g., *Paracoccus marcusii* (Cha et al. 2015), *Acinetobacter* sp. (Huang et al. 2013) and *Pseudomonas stutzeri* (Deng et al. 2014), etc. There are only a few studies in which nitrite was used as sole nitrogen source for denitrifying bacteria. For example, an aerobic heterotrophic denitrifying bacterium *Pseudomonas* sp. YY7 was screened by using nitrite as sole nitrogen source, and its denitrification rate was 18.20 mg NO_2_^−^–N L^−1^day^−1^, and the removal percentage of NO_2_^−^–N was over 80% at initial NO_2_^−^–N concentrations of 10–40 mg/L (Wan et al. 2011). A hypothermia highly efficient nitrite denitrifying bacterium *Pseudomonas putida* Y-12 was isolated and screened using nitrite as sole nitrogen source in a low temperature condition, and the removal percentages of NO_2_^−^–N and total nitrogen (TN) by strain *Pseudomonas putida* Y-12 at 15 °C were 99% and 52%, respectively (He and Li 2015). In this study, strain *Pannonibacter phragmitetus* F1 was isolated and screened from seawall muddy water with NaNO_2_ as sole nitrogen source. Strain *Pannonibacter phragmitetus* F1 can tolerate high concentrations of NaNO_2_, and showed efficient NO_2_^−^–N and NO_3_^−^–N removal abilities when the initial NaNO_2_ concentrations were 0.4–1.6 g/L. The NO_2_^−^–N concentration treated by strain *Pannonibacter phragmitetus* F1 in this study was higher than the NO_2_^−^–N concentrations treated by strain *Pseudomonas* sp. YY7 (10–40 mg/L) and strain *Pseudomonas putida* Y-12 (15.22 mg/L). It was illustrated that strain *Pannonibacter phragmitetus* F1 present a greater potential in denitrification treatment of wastewater with high nitrite concentration.

The tolerance of various denitrifying bacteria to NaNO_2_ concentration is different. If the NaNO_2_ concentration is too low, the supply of nitrogen source required for normal growth of bacteria will be insufficient, affecting their biological activity (Egli and Zinn 2003). However, excessive concentration of NaNO_2_ (50 mg/L) causes serious harm to the growth and normal metabolism of aquatic organisms (Kroupova et al. 2005). Therefore, low or high NaNO_2_ concentration may both affect the denitrification capacity of denitrifying bacteria. Strain *Pannonibacter phragmitetus* F1 can tolerate high concentrations of NaNO_2_ (0.4–1.6 g/L). Furthermore, although the denitrification capacity of strain *Pannonibacter phragmitetus* F1 was significantly reduced (*p* < 0.05) at the initial NO_2_^−^–N concentrations of 2.4 and 3.2 g/L treatments, strain *Pannonibacter phragmitetus* F1 still exhibited an acceptable denitrification capacity (Fig. 6) and there was no mass bacterial death observed. In summary, strain *Pannonibacter phragmitetus* F1 can endure up to 3.2 g/L NaNO_2_, and grew in an acceptable manner and maintained an effective denitrification capacity.

### Growth of strain *Pannonibacter phragmitetus* F1 in neutral and alkaline environments

Most denitrifying bacteria are suitable for growth in neutral and weak alkali environments (Zhang et al. 2012; Ye et al. 2016). The optimum pH of denitrification by most denitrifying bacteria was 7–8 (Tang et al. 2014; He, Li, and Xu 2015; Yang et al. 2013). However, when the pH was out of the optimal range, the denitrification capacity can be decreased. High pH will affect microbial growth and metabolism, leading to the reduction of its denitrification capacity (Zhang et al. 2015; He et al. 2015). The pH can affect the ionization degree of nutrients in the cultivation process, and the ability of bacteria to absorb nutrients is consequently affected, resulting in the weakening of activities of various enzymes in their growth and metabolism (Han et al. 2013). For example, high pH can inhibit the expression of nitrite reductase (*NiRs*), leading to nitrite accumulation in wastewater (Glass and Silverstein 1998). Strain *Pannonibacter phragmitetus* F1 identified in this study can adapt to a wide range of neutral and alkaline environments, and has efficient NO_2_^−^–N and NO_3_^−^–N removal abilities when the initial pH was 7–11. In particular, strain *Pannonibacter phragmitetus* F1 showed the most effective denitrification capacity at the initial pH of 10. The pH of coastal aquaculture wastewater reportedly ranged from 7 to 10 (Li et al. 2018; Yang et al. 2017), demonstrating that strain *Pannonibacter phragmitetus* F1 can be potentially used for nitrogen removal in coastal aquaculture wastewater, especially for locations with high pH (e.g., pH of 10). Furthermore, the alkali-tolerance of strain *Pannonibacter phragmitetus* F1 also makes it possible to be applied in the treatment of drainage water originating from soda saline-alkaline farmlands, which are usually with high pH (Dendooven et al. 2010; Silva et al. 2008).

In this study, the denitrification capacity of strain *Pannonibacter phragmitetus* F1 in saline condition was evaluated considering the influence of pH, initial NaNO_2_ concentration and inoculation size. In saline conditions with 10 g/L NaCl concentration, strain *Pannonibacter phragmitetus* F1 can adapt to a wide range of neutral and alkaline environment and has a promising denitrification capacity. Additionally, strain *Pannonibacter phragmitetus* F1 was tolerant of high concentrations of NaNO_2_ and exhibited efficient nitrogen removal ability with NaNO_2_ concentrations up to 1.6 g/L. The inoculation size is also one of the important factors affecting the biological nitrogen removal of strains. An appropriate inoculation size is of great significance to obtain the highest denitrification capacity of strains. The role of denitrification can not be achieved if the inoculation size is not enough, while, too much inoculation size will cause the biological competition for nutrients and lead to the death of some bacteria (He et al. 2015). In this study, an optimal inoculation size of 3–7% was obtained for strain F1 in regarding to its denitrification capacity. However, other potential influencing factors like the temperature, carbon source and C/N ratio, etc., were not tested. Therefore, the denitrification capacity of strain *Pannonibacter phragmitetus* F1 is recommended to be further evaluated considering the above factors. Furthermore, strain *Pannonibacter phragmitetus* F1 produced a large amount of bubbles in the culture in an anoxic condition as observed in this study. The gases produced in the denitrification process include N_2_, NO and N_2_O in theory (Coban et al. 2015), and it is recommended to quantify these gases in future studies for further revealing the nitrogen conversion mechanism in denitrification process. Furthermore, the practical use of strain *Pannonibacter phragmitetus* F1 for the treatment of real coastal aquaculture wastewater in field conditions is recommended for future study.

## Data Availability

The datasets used and/or analyzed during the current study are available from the corresponding author on reasonable request.
